# New insight on the potential detrimental effect of metabolic syndrome on the Alzheimer disease neuropathology: Mechanistic role

**DOI:** 10.1111/jcmm.70118

**Published:** 2024-12-07

**Authors:** Naif H. Ali, Hayder M. Al‐Kuraishy, Ali I. Al‐Gareeb, Athanasios Alexiou, Marios Papadakis, Mostafa M. Bahaa, Fawaz Alibrahim, Gaber El‐Saber Batiha

**Affiliations:** ^1^ Department of Internal Medicine, Medical College Najran University Najran Saudi Arabia; ^2^ Department of Clinical pharmacology and Medicine, College of Medicine Mustansiriyah University Baghdad Iraq; ^3^ Jabir ibn Hayyan Medical University Najaf Iraq; ^4^ Department of Science and Engineering Novel Global Community Educational Foundation Hebersham New South Wales Australia; ^5^ Department of Research & Development AFNP Med Wien Austria; ^6^ Department of Research & Development Funogen Athens Greece; ^7^ University Centre for Research & Development Chandigarh University Mohali Punjab India; ^8^ Department of Surgery II University Hospital Witten‐Herdecke, University of Witten‐Herdecke Wuppertal Germany; ^9^ Pharmacy Practice Department, Faculty of Pharmacy Horus University New Damietta Egypt; ^10^ Division of Neurology King Abdulaziz Medical City, Ministry of the National Guard Health Affairs Riyadh Saudi Arabia; ^11^ Department of Pharmacology and Therapeutics, Faculty of Veterinary Medicine Damanhour University Damanhour Egypt

**Keywords:** Alzheimer disease, metabolic syndrome, neuroinflammation

## Abstract

The metabolic syndrome or syndrome X is a clustering of different components counting insulin resistance (IR), glucose intolerance, visceral obesity, hypertension and dyslipidemia. It has been shown that IR and dysregulation of insulin signalling play a critical role in the development of metabolic syndrome by initiating the pathophysiology of metabolic syndrome through induction of glucolipotoxicity, impairment of glucose disposal and triggering of pro‐inflammatory response. Furthermore, metabolic syndrome unfavourably affects the cognitive function and the development of different neurodegenerative diseases such as Alzheimer disease (AD) by inducing oxidative stress, neuroinflammation and brain IR. These changes together with brain IR impair cerebrovascular reactivity leading to cognitive impairment. In addition, metabolic syndrome increases the risk for the development of AD. However, the central mechanisms by which metabolic syndrome amplify AD risk are not completely elucidated. Consequently, this narrative review aims to revise from published articles the association between metabolic syndrome and AD regarding cellular and subcellular pathways. In conclusion, metabolic syndrome is regarded as a potential risk factor for the induction of AD neuropathology by different signalling pathways such as initiation of brain IR, activation of inflammatory signalling pathways and neuroinflammation.

## 1 INTRODUCTION

The metabolic syndrome or syndrome X is a clustering of different components counting insulin resistance (IR), glucose intolerance, visceral obesity, hypertension and dyslipidemia.^1^ Syndrome X was first described by Reaven in 1988 who described that IR is not limited in the pathogenesis of type 2 diabetes (T2D) but also intricate in the pathogenesis of cardiovascular diseases.^2^ Reaven suggests that IR is commonly conjugated with other metabolic abnormalities such as visceral obesity, hypertension and dyslipidemia, who described them together as a metabolic syndrome or syndrome X.^3^ Later on, this syndrome was renamed as Reaven's metabolic syndrome to distinguish it from cardiac syndrome X which was initially recognized by Kemp in 1973.^3^ Kemp in an editorial described the undefined chest pain and myocardial ischemia despite of normal coronary angiogram known as cardiac syndrome X.^3^ Moreover, some components of metabolic syndrome were reported previously, for example; Joslin in 1921 and Kylin 1in 1923 described the possible connotation between hypertension, T2D and hyperuricemia.^4^, ^5^ The metabolic syndrome term was reported in literatures since 1967 by Avogadro.^6^ Furthermore, in early 1990s metabolic syndrome was fully described in relation to cardiovascular diseases depending on specific indices including high density lipoprotein (HDL), serum triglyceride (TG), waist circumference and blood glucose.^7^ According to the International Diabetes Federation and American Heart Association, presence of three components of IR, glucose intolerance, visceral obesity, hypertension, and dyslipidemia is considered as diagnostic criteria for the metabolic syndrome.^8^

The fundamental causes of the metabolic syndrome are different including sedentary life, excessive consumption of sweetener beverage and high carbohydrate/fat diet, alcoholism, sleep disorders, aging, chronic stress and genetic factors.^9^ For example, chronic stress can promote the development of metabolic syndrome through dysrgulation of hypothalamic–pituitary axis (HPA) leading to increasing of circulating cortisol level which cause IR, T2D and visceral obesity.^10^ In addition, chronic stress increases night level of cortisol in obesity as evident from animal model studies suggesting that dysregulation of HPA is a contributing factor in the development of metabolic syndrome.^10^ Furthermore, aging enhances obesity and the progression of metabolic syndrome due to the development of skeletal muscle IR.^10^ Notably, aging of skeletal muscles is correlated with increasing of lipid accumulation, oxidative stress, inflammatory changes and mitochondrial dysfunction results in momentous alterations of insulin signalling in the skeletal muscle.^11^ Thus, sarcopenia in elderly patients can induce IR, T2D and the development of metabolic syndrome.^12^

It has been shown that IR and dysregulation of insulin signalling play a critical role in the development of metabolic syndrome by initiating the pathophysiology of metabolic syndrome through induction of glucolipotoxicity, impairment of glucose disposal and triggering of pro‐inflammatory response.^13^ IR triggers central and peripheral metabolic perturbations including brain IR, lipolysis, increasing production of hepatic very low‐density lipoprotein (VLDL), pro‐inflammatory cytokines, inhibition of skeletal glucose uptake, promoting hepatic gluconeogenesis, pancreatic β‐cell dysfunction and peripheral vasoconstriction by suppressing the release of endothelial nitric oxide.^13^ Despite of these findings, there is still debate whether IR is the cause or consequence of metabolic syndrome.

**FIGURE 1:**
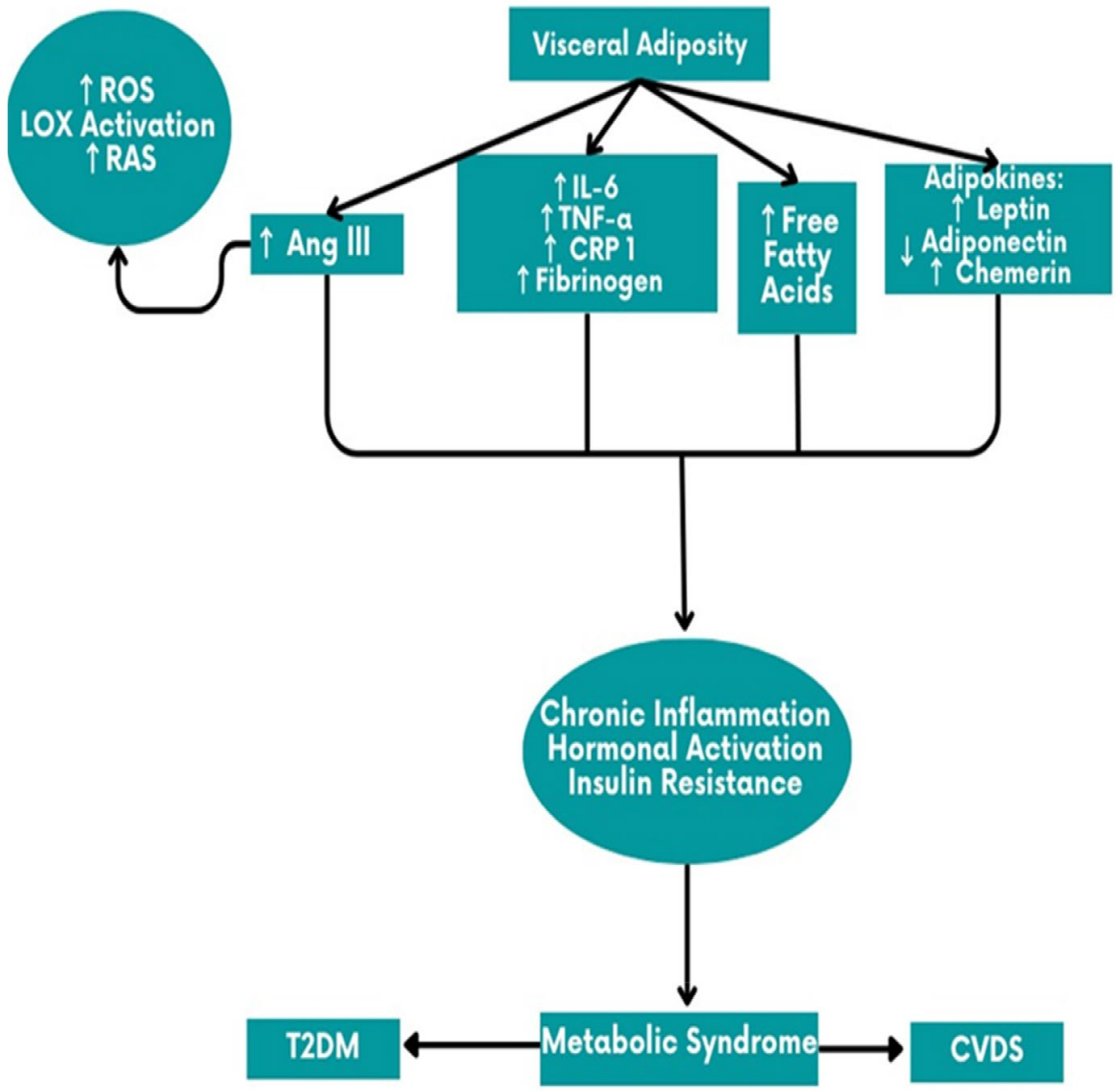
The potential role visceral adiposity in the development of metabolic syndrome.

On the other hand, visceral obesity which is the most prevalent component of the metabolic syndrome seems to be the primary pathology of metabolic syndrome.^14^ However, obesity is regarded as a heterogeneous status, as not every obese patient has cardiometabolic comorbidities.^14^ In particular, visceral obesity have higher hyperlioplytic activity and prothrombotic state increasing risk of cardiovascular complications such as endothelial dysfunction, atherosclerosis and hypertension, and other cardiometabolic derangements such IR and T2D.^15^ Of note, visceral obesity is commonly associated with low‐grade inflammatory condition due to increasing the activity of inflammatory macrophages which induce the release of a pro‐inflammatory cytokine tumour necrosis factor alpha (TNF‐α).^16^ Indeed, low‐grade inflammatory status can cause IR and the development of T2D by inhibiting peripheral insulin signalling and pancreatic β‐cell dysfunction respectively.^17^ Therefore, the pathophysiology of metabolic syndrome is so complex and related to different genetic and environmental factors. However, visceral obesity is the main key factor involved in the pathogenesis of metabolic syndrome by increasing the release of pro‐inflammatory cytokines mainly TNF‐α which intricate in the induction of IR and pathogenesis of T2D, dyslipidemia and hypertension.^18^ Furthermore, visceral obesity through induction of oxidative stress and inflammatory reaction promotes the activation of renin‐angiotensin system (RAS) which implicated in the development of IR and progression of T2D and hypertension.^19^ Visceral adipose tissue has a complete RAS can produced angiotensin II result in systemic inflammation and oxidative stress with subsequent cardiovascular complications.^19^ Of note, RAS is involved in the growth and differentiation of adipocytes, thus; genetic variations in the components of RAS predispose for visceral obesity and hypertension.^20^ Besides, visceral adipose tissue is regarding as a main source of pro‐inflammatory adipocytokines such as chemerin, visfatin and leptin that implicated in the pathogenesis of metabolic syndrome.^21^ Aberrant overexpression and release of adipocytokines in visceral obesity are intricate in the pathogenesis of IR, T2D and hypertension.^21^ Therefore, pro‐inflammatory adipocytokines are considered as a potential link between visceral obesity and other components of metabolic syndrome. In sum, visceral adiposity seems to be the primary event involved in the pathogenesis of metabolic syndrome through different mechanisms (Figure 1).

Moreover, long‐standing untreated metabolic syndrome adversely affects the cognitive function and the development of different neurodegenerative diseases such as Alzheimer disease (AD) and Parkinson disease (PD) by inducing oxidative stress, neuroinflammation and brain IR.^22^ According to neuroimaging studies significant alterations of brain metabolism and white matter architecture has been observed in patients with metabolic syndrome. These changes together with brain IR impair cerebrovascular reactivity leading to cognitive impairment.^23^ In addition, many studies showed that metabolic syndrome increases the risk for the development of AD.^24^, ^25^ However, the fundamental mechanisms by which metabolic syndrome augment AD risk are not fully clarified. Therefore, this narrative review aims to revise from published articles the association between metabolic syndrome and AD regarding cellular and subcellular pathways. In addition, in this review we try to find the most mechanistic pathway linking metabolic syndrome and AD.

## 2 AD NEUROPATHOLOGY

**FIGURE 2:**
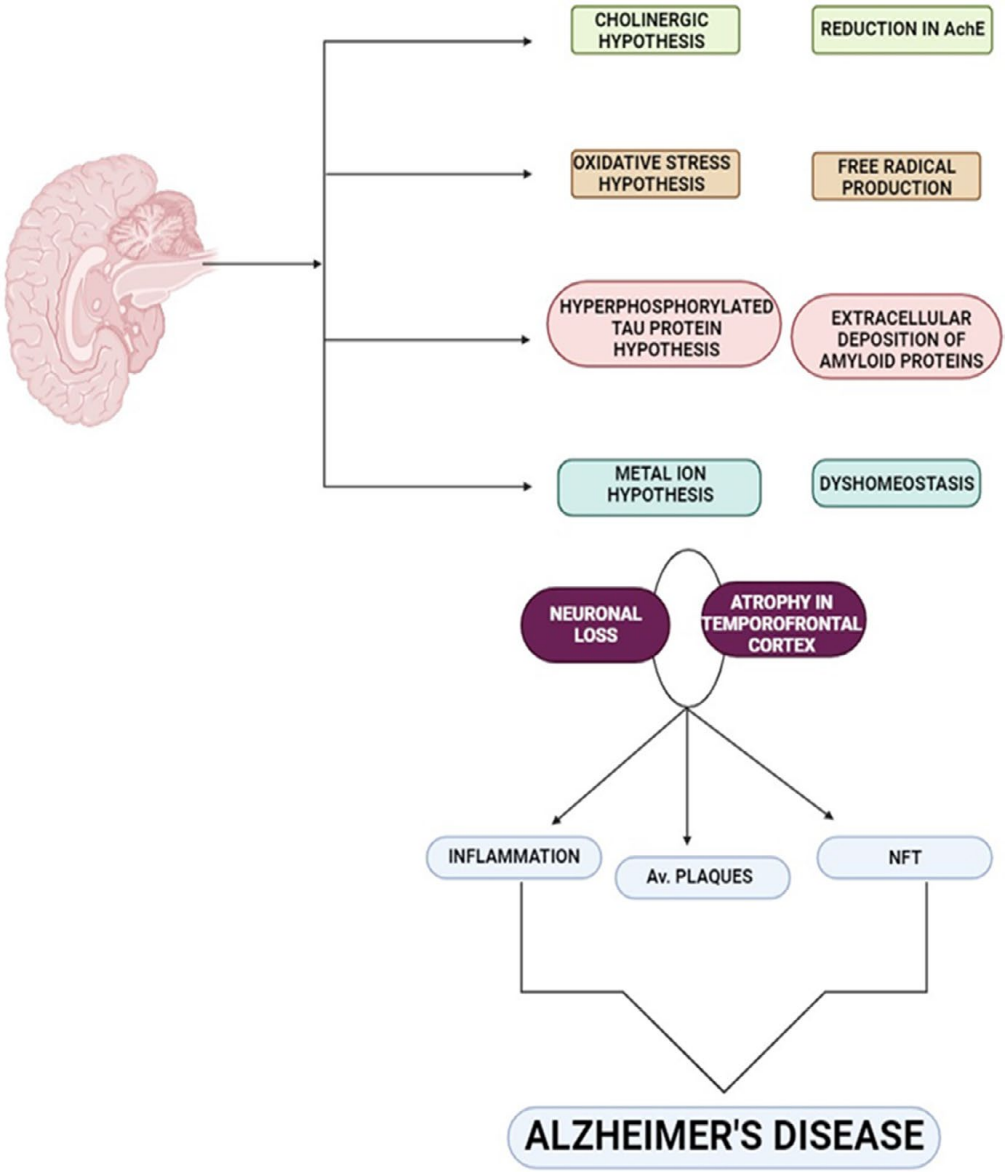
Pathogenesis of Alzheimer's disease (AD).

AD is the most common age‐related neurodegenerative disease implicated in the development and progression of memory decline and cognitive impairment.^26^ AD is more frequent in peoples older than 65 years, and the development of AD in the aged >85 year is called late‐onset AD, though rapid development of AD below age of 65 year is called early‐onset AD.^26^ Most of AD cases are sporadic which account for 90%, and 10% of AD is due to familial causes.^27^ The underlying causes of AD are multifactorial including aging, environmental toxins, malnutrition, head injury and cardiometabolic factors that induce degeneration of cholinergic neurons, which resistible for regulation of memory and cognitive function.^27^ Genetic factors are mainly implicated in the development of early‐onset AD; however environmental factors are mainly involved in the development of late‐onset AD.^28^ Different theories were suggested in the pathogenesis of AD, till now amyloid theory is the most acceptable one.^29^ Pathologic factors in highly susceptible subjects trigger extracellular deposition of amyloid peptide called amyloid beta (Aβ), and intracellular deposition of hyperphosphorylated tau protein with formation of neurofibrillary tangles (NFTs).^27^ Normally, both Aβ and tau proteins are involved in the regulation of the release of neurotransmitters and stability of neuronal microtubules and axonal transport respectively.^30^ In addition, Aβ is eliminated through neuronal autophagy and blood brain barrier by complex mechanism to be metabolized by liver and excreted by kidney. Aβ is generated from amyloid precursor protein (APP) by amyloidogenic pathway which augmented by aging.^31^ Though, in healthy and young subjects, most of APP processing is through non‐amyloidogenic pathway which generates a neuroprotective soluble APP alpha (sAPPα).^32^ Therefore, overproduction of Aβ due to mutation of *APP* gene or defective clearance promotes AD neuropathology by accumulating Aβ which induce hyper‐phosphorylation of tau protein.^32^ Both of insoluble Aβ and hyper‐ phosphorylated tau proteins trigger series of reactions leading to mitochondrial dysfunction, generation of reactive oxygen species (ROS), oxidative stress, endoplasmic reticulum (ER) stress and lipid peroxidation of neuronal membrane.^33^ Remarkably, hyper‐phosphorylated tau protein is more intricate in AD neuropathology than Aβ.^34^ These neuropathological changes provoke synaptic dysfunction and neuronal apoptosis leading to impairment of cognitive function and development of dementia.^35^ Moreover, apolipoprotein E4 (ApoE4) is highly implicated in the pathogenesis of AD.^36^ Notably, ApoE is a functional protein improves the integrity of neurons and synaptic plasticity by reducing oxidative stress.^37^ It has been illustrated that mutation of *ApoE* gene and production of aberrant ApoE can induce overproduction of Aβ and enhance formation of amyloid plaques a hallmark of AD.^37^ ApoE promotes neuronal injury by inducing oxidative stress mainly in familial AD.^38^ As well, ApoE is also interacting with tau protein chiefly in presence of Aβ result in progressive neurodegeneration.^39^ Collectively, AD neuropathology is multifaceted concerning more than single pathway (Figure 2).

## 3 METHOD AND SEARCH STRATEGY

In the present review, database of Scopus, Embase, PubMed and CENTRAL were searched by two independent reviewers to find relevant publications. Screened articles were chosen according to specific eligibility criteria including original articles such as prospective and retrospective studies identifying the causal relationship between metabolic syndrome and AD, unrelated to other diseases, using animal and human studies. The descriptors using MeSH database as follow (Alzheimer disease and Metabolic syndrome), (Alzheimer disease and Insulin resistance), (Alzheimer disease and Obesity), (Alzheimer disease and Hypertension), (Alzheimer disease and Dyslipidemia). All articles according to the inclusion criteria were estimated. However, reviews, letters, articles other than English language were excluded.

## 4 METABOLIC SYNDROME AND AD RISK

It has been illustrated that from different studies that metabolic syndrome could be a potential risk factor for the development and progression of AD. Findings from preclinical studies observed that metabolic syndrome‐induced neuroinflammation of the white matter could be an early sign for the detrimental effect of metabolic syndrome on the cognitive function in AD rat model.^40^ In addition, high‐fat diet can induce poor cognitive performance and may augment AD‐like pathology in animal model studies.^41^, ^42^ Of interest, cerebral vascular alterations induced by metabolic syndrome triggers the development of AD neuropathology by increasing the expression of APP.^43^ Metabolic syndrome in animal model increases stroke risk due to endothelial dysfunction and oxidative stress which induce profound alterations of cerebral microcirculation.[63]. Chronic cerebral ischemia and hypoperfusion enhance AD neuropathology in rat by inducing APP processing in both amyloidogenic and non‐amyliodogenic pathways leading to the formation of Aβ and sAPPα which increased as a compensatory mechanism to reduce Aβ neuropathology.^44^ Notably, exaggerated pro‐inflammatory cytokines in metabolic syndrome induces the formation of Aβ by triggering the expression of *APP* gene in PSV2UTR‐CAT‐transfected cells.^45^ Gonzalez‐Dominguez et al.^46^ found that AD neuropathology in transgenic mice is associated with systemic metabolic disorders as evident by presence of significant changes in different metabolites such as sphingolipids, steroids and acylcarnitines compared to the wild mice. Indeed, systemic alterations such as oxidative stress, bioenergetics failure, impairment of gluconeogenesis and metabolism of branched amino acids are highly present in AD models.^46^ These preclinical observations suggest a bidirectional link between AD and metabolic syndrome.

In clinical setting, a case–control study indicated that AD patients had higher waist circumference and associated with dysregulated metabolic profile characterized by impaired glucose tolerance and dyslipidemia suggesting that metabolic syndrome may be a potential risk factor for the development of AD.^47^ A cross‐sectional study conducted by Hishikawa et al. illustrated that AD patients with metabolic syndrome had greater cognitive impairment compared to AD without metabolic syndrome.^48^ Besides, vascular endothelial dysfunction and brain IR are more profound in AD patients with metabolic syndrome before appearance of brain white matter changes.^48^

It has been shown concluded from a population‐based cohort study that AD risk is more common in women with metabolic syndrome than men^25^ proposing that women are exceedingly vulnerable to the harmful effect of metabolic syndrome. Particularly, metabolic syndrome seems to be highly associated with women compared to men that might due to hormonal changes and risk of gestational diabetes.^49^ Conversely, a large‐population and meta‐analysis study illustrated that metabolic syndrome increases risk of vascular dementia and not linked with AD risk.^50^ It has been revealed that metabolic syndrome triggers the development of cognitive impairment and vascular dementia.[73]. A recent systematic review and meta‐analysis disclosed a significant association between the components of metabolic syndrome and AD incidence.^51^ Likewise, T2D and metabolic syndrome accelerate AD incidence when coexist with mild cognitive impairment, whereas regular use of antidiabetic and antihyperlipidemic agents can reduce AD in patients with metabolic syndrome.^52^ Therefore, lifestyle modification and reduction of cardiovascular risk factors for patients presented with mild cognitive impairment in association with T2D or metabolic syndrome could effective preventive measures to decrease AD risk. These findings highlighted that metabolic syndrome is associated with high AD risk. However, the exact component of metabolic syndrome linked with AD risk was not fully elucidated.

It has been established that high HDL serum level is positively correlated with cognitive performance and memory function.^53^ A cross‐sectional study included 540 subjects aged 68–98 years, free of dementia revealed that subjects with HDL level higher than 60 mg/dL had better cognitive outcomes compared with subjects had low HDL level.^54^ Similarly, a cross‐sectional study observed that subjects with high HDL serum level had better memory performance and good verbal learning.^53^ A prospective cohort study illustrated that HDL serum level > 55 mg/dL is associated with low AD risk.^55^ Importantly, it has been established that decreasing of HDL serum level is implicated in the pathogenesis of AD by inducing abnormal brain lipid metabolism with acceleration formation of Aβ and NFTs.^56^ Of note, CSF‐HDL receptor promotes elimination of soluble Aβ from the brain into the systemic circulation.^57^ Therefore, reduction of HDL serum as in metabolic syndrome could be potential risk factor for the development and progression of AD.

Moreover, hypertriglyceridemia which is an important component of metabolic syndrome is also implicated in AD neuropathology and impairment of cognitive function.^58^ A population‐based cohort study disclosed that moderate hypertriglyceridemia is linked with the development and progression of AD and non‐AD dementia.^59^ Findings from experimental studies demonstrated that TG‐rich lipoproteins enhance the delivery of Aβ from liver to the brain through uptake at CSF level.^60^, ^61^ As well, hypertriglyceridemia can induce cerebral vascular alterations and endothelial dysfunction results in the propagation of dementia neuropathology.^59^ Furthermore, glucose intolerance and hyperglycemia that are commonly associated linked with the metabolic syndrome are also implicated AD neuropathology [85]. Dysregulation of peripheral glucose homeostasis increases AD risk and cognitive impairment. Though, a prospective study showed little role of glucose intolerance in the pathogenesis of AD.^62^ A systematic review and meta‐analysis of preclinical and clinical studies showed that the expression of glucose transporters (GLUT1 and GLUT3) is decreased in cerebral cortex and hippocampus, though GLUT2 and GLUT12 are augmented in cerebral cortex and hippocampus as a compensatory mechanism to reduce brain IR.^63^ Further, systemic hypertension is also intricate in AD neuropathology by increasing buildup of neuronal Aβ.^64^ Notably, development of midlife hypertension is associated with late‐onset AD. A systematic review and meta‐analysis indicated that stage I systolic hypertension increases AD risk by 18% while stage II systolic hypertension increases AD risk by 25%, without association of AD with diastolic hypertension.^65^ Thus, each component of metabolic syndrome contributes in AD neuropathology by different pathways suggesting metabolic‐cognitive syndrome which was proposed by Frisardi et al.^66^

Collectively, these findings highlighted that metabolic syndrome is a possible risk factor for induction and progression of AD by different cellular and molecular mechanism that are not fully interpreted in clinical settings.

## 5 MECHANISTIC LINKS BETWEEN METABOLIC SYNDROME AND AD

### 5.1 Brain IR

**FIGURE 3:**
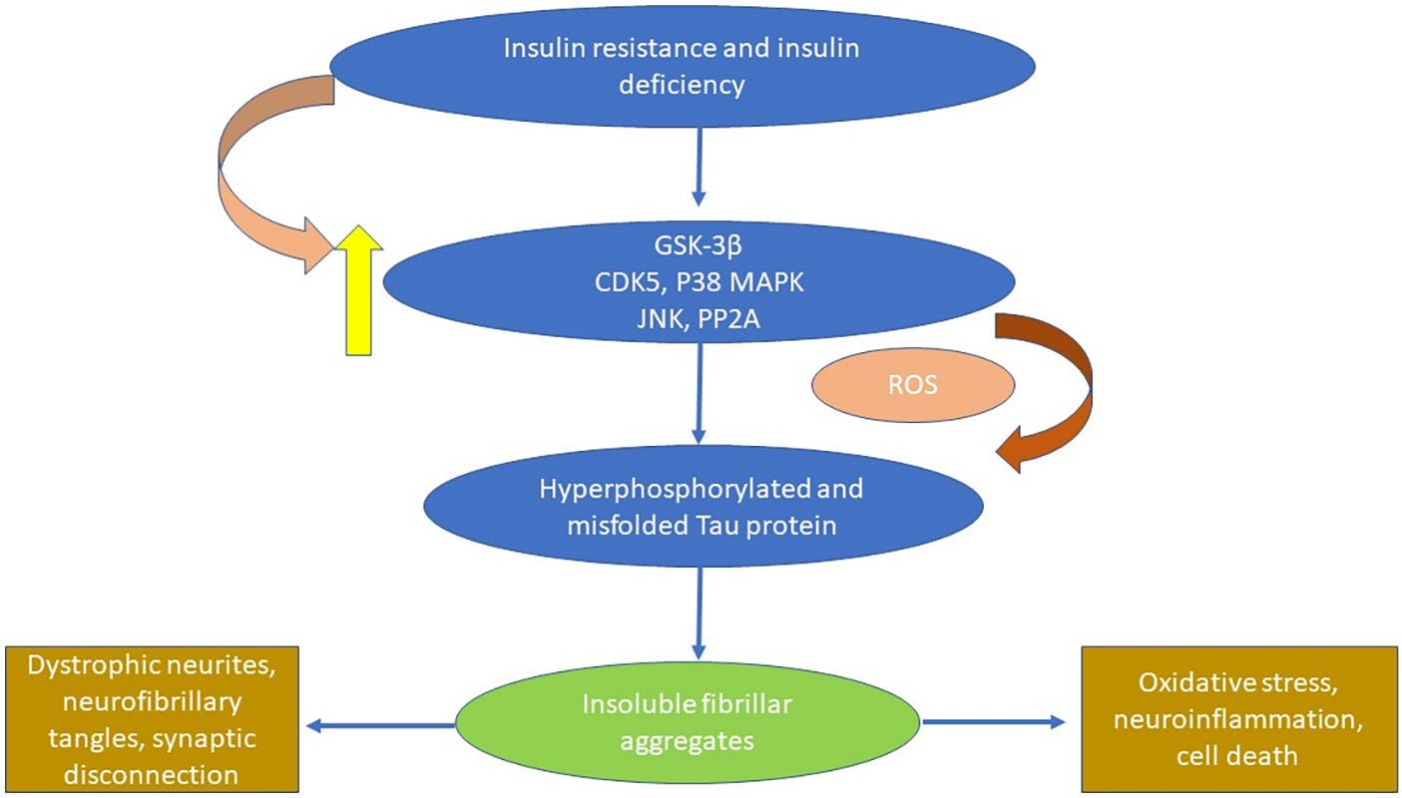
Brain insulin resistance (IR) and Alzheimer disease (AD) neuropathology.

In healthy status, insulin plays a crucial role in regulation of synaptic function; neurogenesis and regulation of neurotransmitter release by inhibiting excitatory glutamatergic neurotransmission and activates cholinergic neurotransmission.^67^ Brain insulin signalling via modulation of glycogen synthase kinase 3 beta (GSK3β) improves neurogenesis.^67^ Yanagita et al. found that dysregulation of brain insulin signalling is linked the development of mood disorders, dementia and neurodegenerative diseases.^67^ Genetic deletion of insulin receptors or insulin receptor substrate in the amygdala and hippocampus results in severe cognitive impairment and deterioration of spatial learning in adult mice.^68^ Brain IR is common feature of different neurodegenerative diseases including AD result in the impairment of hippocampal plasticity and the development of cognitive impairment.^69^ It has been stated that development of metabolic syndrome is associated with the progression of brain IR which cause suppression of hippocampal neurogenesis in animal model study.^70^ Brain IR is often associated with T2D leading neuroinflammation, oxidative stress, neuronal apoptosis and the development of neurodegeneration. As well, peripheral IR in T2D and metabolic disorders can induce brain IR through TNF‐α signalling. However, brain IR may develop independently in AD due to progressive accumulation of Aβ and NFTs regardless of ApoE4 status or peripheral blood glucose.^71^ In addition, brain IR promotes buildup of Aβ and NFTs, and inhibits their clearance. Thus, brain IR is developed in early AD and contributes in progressive neurodegeneration. Furthermore, brain IR results in abnormal neuronal glucose metabolism and neuronal energy leading to cognitive impairment and memory loss.^72^ According to these findings, AD was proposed as type 3 diabetes (T3D) due to abnormal insulin signalling and glucose metabolism in the brain.^73^ Different studies disclosed that brain IR plays a critical role in the pathogenesis of AD by inducing APP expression, hyperphosphorylation of tau protein, neuronal oxidative stress, mitochondrial dysfunction, ER stress, and the development of neuroinflammation.^74^, ^75^ Likewise, brain IR suppresses specific genes intricate in the regulation of synaptic plasticity and cholinergic neurotransmission causing neurocognitive impairment. Moreover, brain IR triggers the expression of GSK3β, protein phosphatase 2 A (PP2A), cyclin‐dependent kinase 5 (Cdk5), p38 mitogen activated protein kinase (p38MAPK), and Janus N‐terminal kinase (JNK) that increase hyperphosphorylation of tau protein which cause neuroinflammation, oxidative stress and neuronal injury^76^ (Figure 3).

**FIGURE 4:**
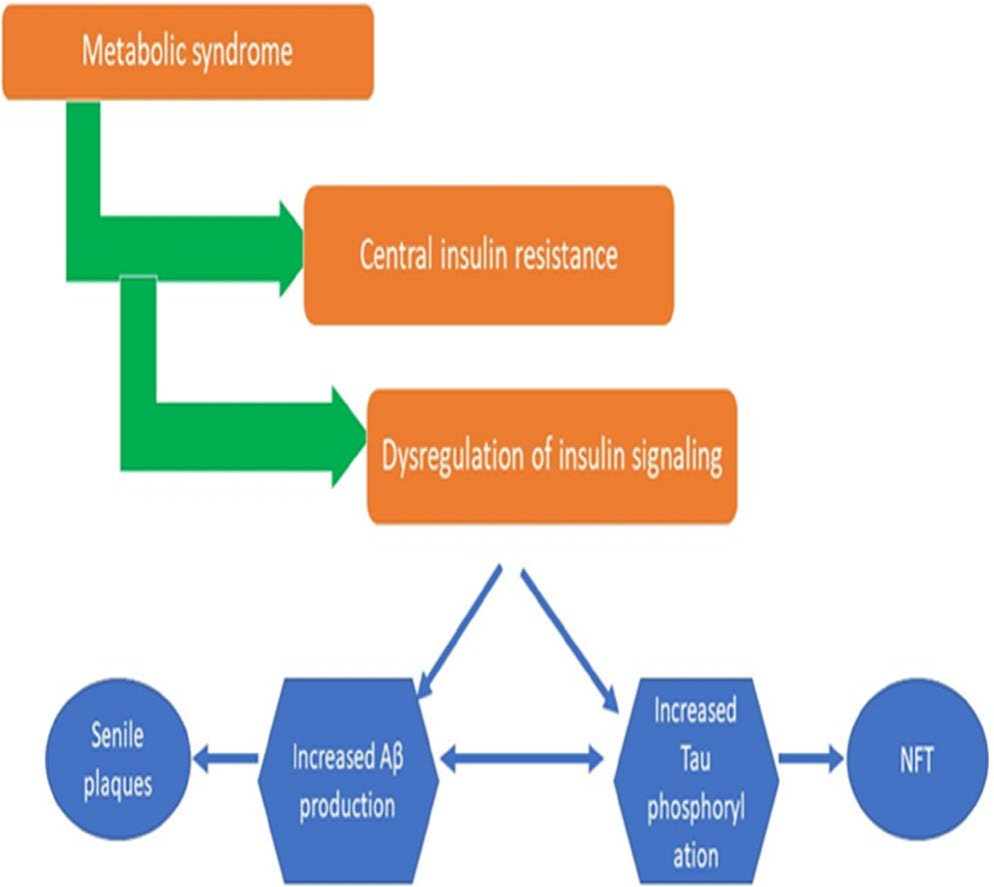
Metabolic syndrome and risk of brain insulin resistance (IR).

On the other hand, metabolic syndrome via chronic low‐grade inflammatory status can induce peripheral IR. In a similar manner, chronic low‐grade inflammatory disorders and high level of TNF‐α in the metabolic syndrome may induce the development of brain IR.^77^ A population study disclosed that brain structure and volume assessed by PET and MRI are reduced in patients with metabolic syndrome.^77^ White matter volume and brain parenchyma fractions are distorted in patients with metabolic syndrome due to the development of brain IR.^78^ Therefore, metabolic syndrome through induction of brain IR can trigger AD neuropathology by increasing Aβ formation and hyperphosphorylation of tau protein leading to the generation of amyloid plaques and NFTs respectively (Figure 4).

### 5.2 GSK3β

GSK3 is a threonine serine protein kinase controls glycogen synthesis and involved in the regulation of blood glucose and cellular metabolism.^79^ The most notorious GSK3 type is GSK3β which highly expressed in the brain is involved in the regulation of synaptic plasticity and neurogenesis through PI3K and Wnt/β‐catenin signalling pathways.^80^ GSK3β is also involved in induction of neuronal oxidative stress by inhibiting the expression of nuclear factor erythroid 2 related factor 2 (Nrf2).^80^

**FIGURE 5:**
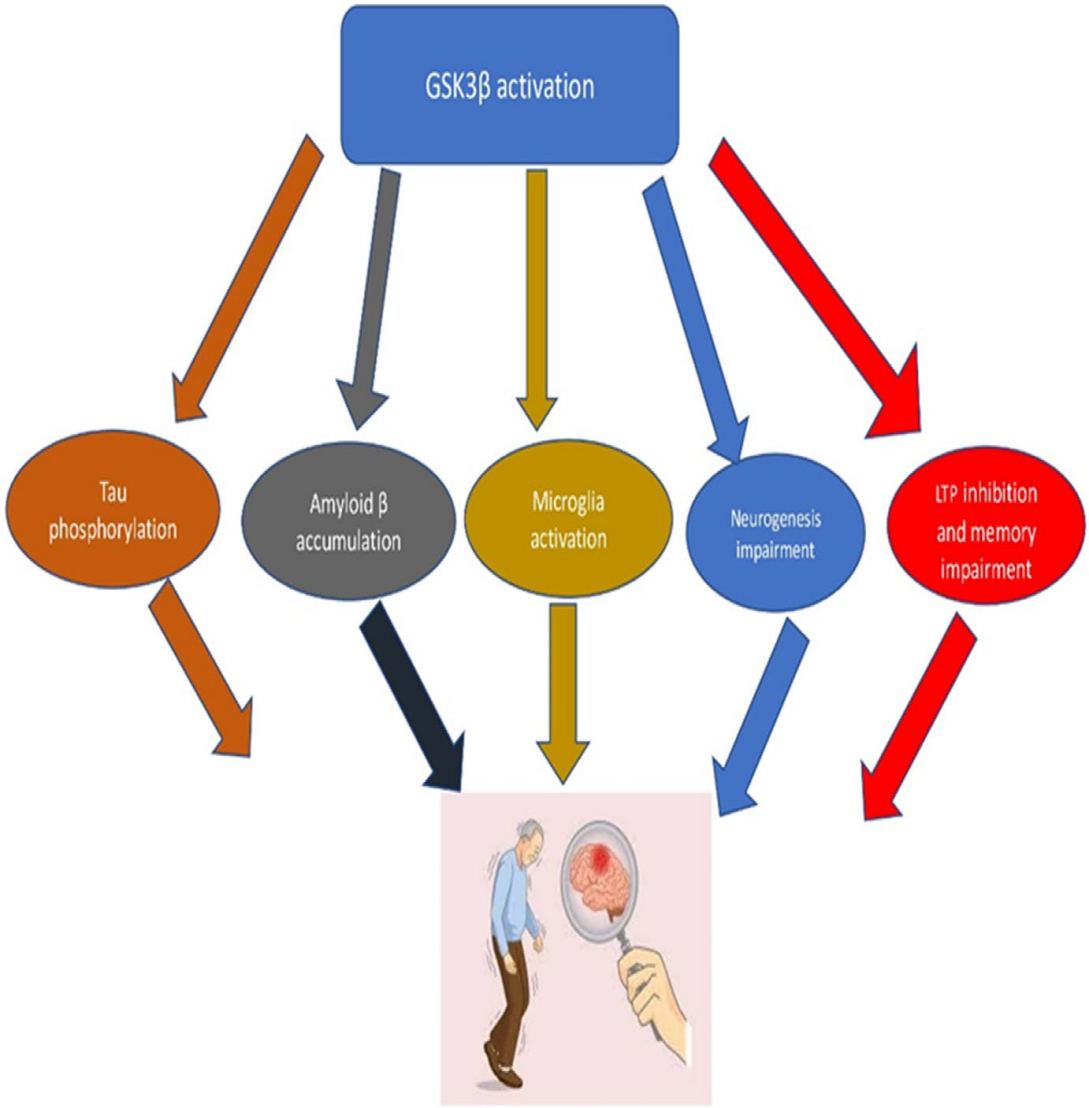
GSK3β overactivity and Alzheimer disease (AD) neuropathology.

It has been shown that GSK3β is suppressed by insulin and insulin‐like growth factor 1 (IGF‐1) via activation of protein kinase A (PKA). Brain IR and dysregulation of neuronal insulin signalling induce GSK3β [112]. Findings from preclinical studies showed that high‐fat diet (HFD)‐induced cognitive impairment in mice is mediated by triggering neuronal GSK3β activity which causes oxidative stress, mitochondrial dysfunction and neuroinflammation.^81^ Of interest, the expression and activity of GSK3β are augmented in the brains AD patients mainly in the frontal cortex.^82^ Remarkably, GSK3β overactivity is started before deposition of NFTs suggesting that GSK3β might be a primary event involved in AD neuropathology.^82^ In addition, GSK3β activity in the platelets is augmented and correlated with cognitive impairment and disease severity in AD patients.^83^ Besides, exaggeration of neuronal GSK3β activity is associated with Aβ accumulation and tau protein hyperphosphorylation through induction of APP processing and neuronal oxidative stress.^84^ As well, GSK3β overactivity is associated with microglial activation and inhibition of neurogenesis result in cognitive impairment^84^ (Figure 5).

Moreover, neuronal GSK3β overactivity may be develop due to IR, T2D and obesity. GSK3β overactivity is associated with visceral obesity to control differentiation of pre‐adipocytes. HFD‐induced metabolic syndrome in rats is mediated by activating GSK3β which reduce insulin sensitivity, and using of GSK3β inhibitor lithium can reverse these metabolic alterations.^85^ Inhibition of brain GSK3β improves neuronal insulin substrate 1 (IRS‐1) which reduced central sympathetic outflow and decrease blood pressure proposing that aberrant GSK3β expression in the brain is implicated in the pathogenesis of central hypertension.^86^

These findings indicated that metabolic syndrome may induce AD neuropathology through triggering of neuronal GSK3β overactivity.

### 5.3 Oxidative stress and mitochondrial dysfunction

Of note, oxidative stress and mitochondrial dysfunction are often involved in the pathogenesis of AD leading to lipid peroxidation of neuronal membrane and impairment of neuronal energy homeostasis.^87^ It has been revealed that biomarkers of mitochondrial dysfunction and oxidative stress are augmented in the CSF and other brain areas in AD. Additionally, oxidative stress and mitochondrial dysfunction promote AD neuropathology by increasing APP processing and generation of neurotoxic Aβ which also induce the development and progression of neuronal mitochondrial dysfunction and oxidative stress.^88^ Furthermore, oxidative stress and mitochondrial dysfunction may induce AD neuropathology indirectly by reducing the expression of the neuroprotective SIRT1 which improve neurogenesis and reduce neuroinflammation in different neurodegenerative diseases.^89^ It has been established that neuronal SIRT1 signalling is extremely reduced in AD due to oxidative stress and mitochondrial dysfunction.^90^ As well, neurotoxic Aβ‐induced oxidative stress and mitochondrial dysfunction can trigger neuronal apoptosis by inducing apoptotic p53 and reducing SIRT1 signalling.^90^ Therefore, antioxidant agents could be of therapeutic value in the management of neurodegenerative diseases including AD. As well, modulation of endogenous antioxidant defence mechanism is an integral method to reduce oxidative and improve cell stress response in neurodegenerative diseases by increasing the expression of SIRT1 and Nrf2.^90^ In addition, regulation of brain NO‐cGMP signalling can reduce oxidative stress and mitochondrial dysfunction through inhibition of neurotoxicity.^90^ Thus, stimulation of vitagenes against oxidative stress could be a novel therapeutic strategy against cancer and neurodegenerative diseases.^91^ During chronic alcohol intoxication DNA damage observed in the cerebellum and hippocampus was correlated with oxidative stress, mitochondrial dysfunction, and lipid peroxidation.^92^ Thus, activating of brain antioxidant enzymes may reduce oxidative stress and mitochondrial dysfunction that linked with neurodegeneration in AD.

Furthermore, oxidative stress and mitochondrial dysfunction are often associated in the pathophysiology of metabolic syndrome.^92^ These cellular changes in the metabolic syndrome can induced the development of brain IR and progression of AD.^92^ Peripheral IR in the metabolic syndrome triggers lipolysis and increasing circulating free‐fatty acids which promote neuronal oxidative stress and mitochondrial dysfunction by inhibiting neuronal insulin signalling.^93^ Indeed, metabolic syndrome‐induced oxidative stress and mitochondrial dysfunction reduce the expression of neuronal GLUT4.^94^ Notably, SIRT1 signalling is highly reduced in the metabolic syndrome and activation of SIRT1 can protect against metabolic syndrome‐induced neurodegeneration.^95^ Likewise, metabolic syndrome‐induced oxidative stress and mitochondrial dysfunction inhibits the expression of peripheral and central Nrf2.^96^ A previous experimental study demonstrated that deletion of *Nrf2* gene results in the development of severe metabolic syndrome in mice [135]. Reduction of Nrf2 in visceral obesity inhibits insulin signalling leading to the development of peripheral IR and glucose intolerance.^97^ Nrf2 has a potent neuroprotective role against neurodegenerative diseases and improve cognitive function in AD.^97^ Genetic deletion of *Nrf2* gene in transgenic mice overexpressing APP triggers severe cognitive dysfunction and impairment of spatial learning ability due to progressive accumulation of Aβ and associated synaptic failure.^97^ Preclinical and clinical findings showed that phosphorylated Nrf2 is increased in human peripheral blood cells of AD patients and in AD mouse model at various stages as a compensatory mechanism to reduce oxidative stress in AD.^98^

In sum, oxidative stress and mitochondrial dysfunction together with dysregulation of different signalling pathways such as SIRT1 and Nrf2 adversely affect AD neuropathology.

### 5.4 Inflammatory signalling pathways

Of note, inflammatory signalling pathways such as p38MAPK, nod‐like receptor pyrin 3 (NLRP3) inflammasome and nuclear factor kappa B (NF‐κB) are intricate in the pathogenesis of various types of neurodegenerative diseases.^99^ It has been revealed that NF‐κB signalling is augmented in AD patients compared to healthy controls.^99^ Exaggeration of inflammatory signalling pathways in AD is due to Aβ and NFTs which through oxidative stress promote the expression of NF‐κB.^100^ For example, NFTs trigger the expression of NLRP3 inflammasome in primary cortical neurons.^101^ Halle et al. observed that NLRP3 inflammasome is released in response to the effect of Aβ which induce the expression and release of IL‐1β.^102^ Notoriously, expression of NLRP3 inflammasome precedes onset of Aβ and NFTs accumulation. Likewise, p38MAPK is also implicated in the pathogenesis of AD by enhancing the accumulation of Aβ and NFTs. As well, Aβ and NFTs provoke neuronal p38MAPK signalling.^103^

On the other hand, inflammatory signalling pathways are highly exaggerated in the metabolic syndrome.^104^ For example, nutrient excess and low‐grade inflammatory reactions in the metabolic syndrome provoke the activation of NF‐κB, p38MAPK and NLRP3 inflammasome signalling.^105^ Thus, inflammatory signalling pathways which are upregulated in the metabolic syndrome may induce AD neuropathology through propagation of systemic inflammation.

### 5.5 Neuroinflammation

Neuroinflammation is a specific immune response of the CNS to the stress or exogenous stimuli. Acute neuroinflammation has a neuroprotective effect by eliminating the causative factors, though chronic neuroinflammation with overactivity of astrocytes and microglia provokes progressive neurodegeneration by inducing derangement of BBB and inhibiting of neuronal homeostasis.^106^ It has been observed that neuroinflammation exacerbates AD neuropathology by different mechanisms such as induction of synaptic failure, inhibition of hippocampal neurogenesis and triggering of neuronal apoptosis.^107^ In transgenic mice overexpressing APP/PS1, neuroinflammation and exaggerated inflammatory signalling pathways result in acute cognitive impairment through dysregulation of hippocampal synaptic plasticity.^107^ A cohort study illustrated that biomarker of neuroinflammation in the CSF such as IL‐6, IL‐15 and MCP‐1 are increased in AD patients with cognitive impairment compared to AD without cognitive impairment.^108^ These findings highlighted that neuroinflammation is regarded as a potential mechanism intricate in the pathogenesis of AD.

Furthermore, metabolic syndrome through low‐grade inflammatory disorders and activated inflammatory signalling may induce neuroinflammation. Of note, IR, hyperglycemia, visceral obesity, hypertension and dyslipidemia are often associated with systemic low‐grade inflammatory disorders which induced neuroinflammation.^109^ In addition, IR and leptin resistance are linked with the development of neuroinflammation.^110^ Thus, metabolic syndrome‐induced neuroinflammation could the most important mechanistic pathway in the development and progression of AD.

### 5.6 Hydrogen sulfide

Hydrogen sulfide (H2S) is a messenger molecule modulates myriad signalling cascades and has been conserved across evolutionary boundaries. Although traditionally known as an environmental toxin, H2S is also synthesized endogenously to exert modulatory and homeostatic effects in a broad array of physiologic functions.^111^ H2S levels are tightly physiologically regulated, as both its excess and low levels can be toxic (hormesis).^112^, ^113^ Certainly, at high concentrations, H_2_S inhibits cytochrome c oxidase and uncouples oxidative phosphorylation, which decreases ATP production though; at lower concentrations, H_2_S has stimulatory and protective effects on mitochondria.^113^ Accumulating evidence has revealed pivotal roles for H2S in neuroprotection and normal cognitive function, and H2S homeostasis is dysregulated in neurodegenerative conditions.^114^ H2S production in the course of methionine and cysteine catabolism and its degradation are finely balanced, and impairment of H_2_S homeostasis is associated with various pathologies. Despite the strong geroprotective action of exogenous H_2_S, there are controversial effects of H_2_S and its donors on longevity in other models, as well as on stress resistance, age‐related pathologies, and aging processes.^113^ In AD, the H2S level is reduced due to disturbance of cystathionine β‐synthase (CBS) which synthesis H2S. Reduction of plasma H_2_S level was detected in the plasma of AD patients and correlated with severity of disease.^115^ Reduced H2S that correlated with brain energy levels in the APP/PS1 mouse model of AD was additionally demonstrated.^116^ Findings from preclinical studies highlighted that the fast release H_2_S donor sodium hydrosulfide attenuate neuroinflammation by inhibiting the release of pro‐inflammatory cytokines.^117^ In addition, sodium hydrosulfide prevents Aβ‐induced toxicity in microglial cultures.^118^

On the other hand, H2S which mediate the vasodilatory effect in the mediator in the cardiovascular system is reduced in metabolic syndrome. A lack of H2S and its synthesizing enzyme, cystathionine γ‐lyase, in the vasculature causes hypertension and IR.^119^ A recent experimental study revealed that high‐fat diet‐induced metabolic syndrome is associated with reduction of circulating H2S level in rat model.^120^ Diminished H2S levels in both plasma and tissue contribute in the the development of vascular dysfunction and progression of metabolic syndrome.^120^ These findings highlighted that low H2S level in metabolic syndrome predisposes for the development of AD neuropathology.

Taken together, metabolic syndrome is regarded as a potential risk factor for the induction of AD neuropathology by different signalling pathways such as induction of brain IR, activation of inflammatory signalling pathways and neuroinflammation.

## 6 CONCLUSIONS

The metabolic syndrome may adversely affect the cognitive function and the development of AD by inducing oxidative stress, neuroinflammation and brain IR. These changes together with brain IR impair cerebrovascular reactivity leading to cognitive impairment. Metabolic syndrome through induction of brain IR can trigger AD neuropathology by increasing Aβ formation and hyperphosphorylation of tau protein leading to the generation of amyloid plaques and NFTs respectively. Oxidative stress and mitochondrial dysfunction together with dysregulation of different signalling pathways such as SIRT1 and Nrf2 adversely affect AD neuropathology. Furthermore, inflammatory signalling pathways which are upregulated in the metabolic syndrome may induce AD neuropathology through propagation of systemic inflammation. As well, metabolic syndrome‐induced neuroinflammation could the most important mechanistic pathway in the development and progression of AD.

Taken together, metabolic syndrome is observed as a potential risk factor for the induction of AD neuropathology by diverse signalling pathways such as induction of brain IR, activation of inflammatory signalling pathways and neuroinflammation. This review cannot give the final decision regarding the link between metabolic syndrome and AD, therefore; large‐scale prospective and longitudinal are recommended in this regard.

## AUTHOR CONTRIBUTIONS

**Naif H. Ali:** Writing – review and editing (equal). **Hayder M. Al‐Kuraishy:** Conceptualization (equal); data curation (equal); formal analysis (equal); funding acquisition (equal); investigation (equal); methodology (equal); project administration (equal); resources (equal); software (equal); supervision (equal); validation (equal); visualization (equal); writing – original draft (equal); writing – review and editing (equal). **Ali I. Al‐Gareeb:** Conceptualization (equal); data curation (equal); formal analysis (equal); funding acquisition (equal); investigation (equal); methodology (equal); project administration (equal); resources (equal); software (equal); supervision (equal); validation (equal); visualization (equal); writing – original draft (equal); writing – review and editing (equal). **Athanasios Alexiou:** Conceptualization (equal); data curation (equal); formal analysis (equal); funding acquisition (equal); investigation (equal); methodology (equal); project administration (equal); resources (equal); software (equal); supervision (equal); validation (equal); visualization (equal); writing – original draft (equal); writing – review and editing (equal). **Marios Papadakis:** Conceptualization (equal); data curation (equal); formal analysis (equal); funding acquisition (equal); investigation (equal); methodology (equal); project administration (equal); resources (equal); software (equal); supervision (equal); validation (equal); visualization (equal); writing – original draft (equal); writing – review and editing (equal). **Mostafa M. Bahaa:** Conceptualization (equal); data curation (equal); formal analysis (equal); funding acquisition (equal); investigation (equal); methodology (equal); project administration (equal); resources (equal); software (equal); supervision (equal); validation (equal); visualization (equal); writing – original draft (equal); writing – review and editing (equal). **Fawaz Alibrahim:** Writing – review and editing (equal). **Gaber El‐Saber Batiha:** Conceptualization (equal); data curation (equal); formal analysis (equal); funding acquisition (equal); investigation (equal); methodology (equal); project administration (equal); resources (equal); software (equal); supervision (equal); validation (equal); visualization (equal); writing – original draft (equal); writing – review and editing (equal).

## FUNDING INFORMATION

Open access funding enabled and organized by Project DEAL. This work was supported by University of Written‐Herdeck, Germany.

## CONFLICT OF INTEREST STATEMENT

The authors declare no competing interests.

## Data Availability

All data are available in the manuscript.
